# Supplementary material for: Resolving the full spectrum of human genome variation using Linked-Reads
Source: Genome Res. 2019 Apr;29(4):635–45. doi: 10.1101/gr.234443.118 (PMC6442396; doi:10.1101/gr.234443.118)
Supplement: Supplemental Material [file supp_gr.234443.118_Supplemental_longranger-2.2.zip › longranger-2.2.tar.bz2!/lr2.2-public/lib/stan/cmdstan/stan/doc/api/html/classstan_1_1optimization_1_1_b_f_g_s_line_search.html]

Stan: stan::optimization::BFGSLineSearch< M, QNUpdateType, Scalar, DimAtCompile > Class Template Reference


|  |  |
| --- | --- |
|  | Stan  2.5.0  probability, sampling & optimization |


- Main Page
- Related Pages
- Namespaces
- Classes
- Files

- Class List
- Class Index
- Class Hierarchy
- Class Members

All Classes Namespaces Files Functions Variables Typedefs Enumerations Enumerator Friends Macros Pages

- stan
- optimization
- BFGSLineSearch

Public Types |
Public Member Functions |
List of all members

stan::optimization::BFGSLineSearch< M, QNUpdateType, Scalar, DimAtCompile > Class Template Reference

`#include <bfgs.hpp>`

Inheritance diagram for stan::optimization::BFGSLineSearch< M, QNUpdateType, Scalar, DimAtCompile >:

|  |  |
| --- | --- |
| Public Types | |
| typedef BFGSMinimizer  < ModelAdaptor< M >  , QNUpdateType, Scalar,   DimAtCompile > | BFGSBase |
|  | |
| typedef BFGSBase::VectorT | vector\_t |
|  | |
| typedef stan::math::index\_type  < vector\_t >::type | idx\_t |
|  | |
| Public Types inherited from stan::optimization::BFGSMinimizer< ModelAdaptor< M >, QNUpdateType, Scalar, DimAtCompile > | |
| typedef Eigen::Matrix< Scalar,   DimAtCompile, 1 > | VectorT |
|  | |
| typedef Eigen::Matrix< Scalar,   DimAtCompile, DimAtCompile > | HessianT |
|  | |

|  |  |
| --- | --- |
| Public Member Functions | |
|  | BFGSLineSearch (M &model, const std::vector< double > &params\_r, const std::vector< int > &params\_i, std::ostream \*msgs=0) |
|  | |
| void | initialize (const std::vector< double > &params\_r) |
|  | |
| size\_t | grad\_evals () |
|  | |
| double | logp () |
|  | |
| double | grad\_norm () |
|  | |
| void | grad (std::vector< double > &g) |
|  | |
| void | params\_r (std::vector< double > &x) |
|  | |
| Public Member Functions inherited from stan::optimization::BFGSMinimizer< ModelAdaptor< M >, QNUpdateType, Scalar, DimAtCompile > | |
| QNUpdateType & | get\_qnupdate () |
|  | |
| const QNUpdateType & | get\_qnupdate () const |
|  | |
| const Scalar & | curr\_f () const |
|  | |
| const VectorT & | curr\_x () const |
|  | |
| const VectorT & | curr\_g () const |
|  | |
| const VectorT & | curr\_p () const |
|  | |
| const Scalar & | prev\_f () const |
|  | |
| const VectorT & | prev\_x () const |
|  | |
| const VectorT & | prev\_g () const |
|  | |
| const VectorT & | prev\_p () const |
|  | |
| Scalar | prev\_step\_size () const |
|  | |
| Scalar | rel\_grad\_norm () const |
|  | |
| Scalar | rel\_obj\_decrease () const |
|  | |
| const Scalar & | alpha0 () const |
|  | |
| const Scalar & | alpha () const |
|  | |
| const size\_t | iter\_num () const |
|  | |
| const std::string & | note () const |
|  | |
| std::string | get\_code\_string (int retCode) |
|  | |
|  | BFGSMinimizer (ModelAdaptor< M > &f) |
|  | |
| void | initialize (const VectorT &x0) |
|  | |
| int | step () |
|  | |
| int | minimize (VectorT &x0) |
|  | |

|  |  |
| --- | --- |
| Additional Inherited Members | |
| Public Attributes inherited from stan::optimization::BFGSMinimizer< ModelAdaptor< M >, QNUpdateType, Scalar, DimAtCompile > | |
| LSOptions< Scalar > | \_ls\_opts |
|  | |
| ConvergenceOptions< Scalar > | \_conv\_opts |
|  | |
| Protected Attributes inherited from stan::optimization::BFGSMinimizer< ModelAdaptor< M >, QNUpdateType, Scalar, DimAtCompile > | |
| ModelAdaptor< M > & | \_func |
|  | |
| VectorT | \_gk |
|  | |
| VectorT | \_gk\_1 |
|  | |
| VectorT | \_xk\_1 |
|  | |
| VectorT | \_xk |
|  | |
| VectorT | \_pk |
|  | |
| VectorT | \_pk\_1 |
|  | |
| Scalar | \_fk |
|  | |
| Scalar | \_fk\_1 |
|  | |
| Scalar | \_alphak\_1 |
|  | |
| Scalar | \_alpha |
|  | |
| Scalar | \_alpha0 |
|  | |
| size\_t | \_itNum |
|  | |
| std::string | \_note |
|  | |
| QNUpdateType | \_qn |
|  | |

## Detailed Description

### template<typename M, typename QNUpdateType, typename Scalar = double, int DimAtCompile = Eigen::Dynamic> class stan::optimization::BFGSLineSearch< M, QNUpdateType, Scalar, DimAtCompile >

Definition at line 394 of file bfgs.hpp.

## Member Typedef Documentation

template<typename M , typename QNUpdateType , typename Scalar = double, int DimAtCompile = Eigen::Dynamic>

typedef BFGSMinimizer<ModelAdaptor<M>,QNUpdateType,Scalar,DimAtCompile> stan::optimization::BFGSLineSearch< M, QNUpdateType, Scalar, DimAtCompile >::BFGSBase

Definition at line 398 of file bfgs.hpp.

template<typename M , typename QNUpdateType , typename Scalar = double, int DimAtCompile = Eigen::Dynamic>

typedef stan::math::index\_type<vector\_t>::type stan::optimization::BFGSLineSearch< M, QNUpdateType, Scalar, DimAtCompile >::idx\_t

Definition at line 400 of file bfgs.hpp.

template<typename M , typename QNUpdateType , typename Scalar = double, int DimAtCompile = Eigen::Dynamic>

typedef BFGSBase::VectorT stan::optimization::BFGSLineSearch< M, QNUpdateType, Scalar, DimAtCompile >::vector\_t

Definition at line 399 of file bfgs.hpp.

## Constructor & Destructor Documentation

template<typename M , typename QNUpdateType , typename Scalar = double, int DimAtCompile = Eigen::Dynamic>

|  |  |  |  |
| --- | --- | --- | --- |
| stan::optimization::BFGSLineSearch< M, QNUpdateType, Scalar, DimAtCompile >::BFGSLineSearch | ( | M & | *model*, |
|  |  | const std::vector< double > & | *params\_r*, |
|  |  | const std::vector< int > & | *params\_i*, |
|  |  | std::ostream \* | *msgs* = `0` |
|  | ) |  |  |

inline

Definition at line 402 of file bfgs.hpp.

## Member Function Documentation

template<typename M , typename QNUpdateType , typename Scalar = double, int DimAtCompile = Eigen::Dynamic>

|  |  |  |  |  |  |
| --- | --- | --- | --- | --- | --- |
| void stan::optimization::BFGSLineSearch< M, QNUpdateType, Scalar, DimAtCompile >::grad | ( | std::vector< double > & | *g* | ) |  |

inline

Definition at line 423 of file bfgs.hpp.

template<typename M , typename QNUpdateType , typename Scalar = double, int DimAtCompile = Eigen::Dynamic>

|  |  |  |  |  |
| --- | --- | --- | --- | --- |
| size\_t stan::optimization::BFGSLineSearch< M, QNUpdateType, Scalar, DimAtCompile >::grad\_evals | ( |  | ) |  |

inline

Definition at line 420 of file bfgs.hpp.

template<typename M , typename QNUpdateType , typename Scalar = double, int DimAtCompile = Eigen::Dynamic>

|  |  |  |  |  |
| --- | --- | --- | --- | --- |
| double stan::optimization::BFGSLineSearch< M, QNUpdateType, Scalar, DimAtCompile >::grad\_norm | ( |  | ) |  |

inline

Definition at line 422 of file bfgs.hpp.

template<typename M , typename QNUpdateType , typename Scalar = double, int DimAtCompile = Eigen::Dynamic>

|  |  |  |  |  |  |
| --- | --- | --- | --- | --- | --- |
| void stan::optimization::BFGSLineSearch< M, QNUpdateType, Scalar, DimAtCompile >::initialize | ( | const std::vector< double > & | *params\_r* | ) |  |

inline

Definition at line 412 of file bfgs.hpp.

template<typename M , typename QNUpdateType , typename Scalar = double, int DimAtCompile = Eigen::Dynamic>

|  |  |  |  |  |
| --- | --- | --- | --- | --- |
| double stan::optimization::BFGSLineSearch< M, QNUpdateType, Scalar, DimAtCompile >::logp | ( |  | ) |  |

inline

Definition at line 421 of file bfgs.hpp.

template<typename M , typename QNUpdateType , typename Scalar = double, int DimAtCompile = Eigen::Dynamic>

|  |  |  |  |  |  |
| --- | --- | --- | --- | --- | --- |
| void stan::optimization::BFGSLineSearch< M, QNUpdateType, Scalar, DimAtCompile >::params\_r | ( | std::vector< double > & | *x* | ) |  |

inline

Definition at line 429 of file bfgs.hpp.

---

The documentation for this class was generated from the following file:

- src/stan/optimization/bfgs.hpp


---

[ Stan Home Page ]

*© 2011–2014,
Stan Development Team.*
